# The optical, seismic, and infrasound signature of the March 5 2022, bolide over Central Italy

**DOI:** 10.1038/s41598-023-48396-8

**Published:** 2023-11-30

**Authors:** Marco Olivieri, Davide Piccinini, Gilberto Saccorotti, Dario Barghini, Daniele Gardiol, Nicola Alessandro Pino, Maurizio Ripepe, Giulio Betti, Giorgio Lacanna, Lorenzo Arcidiaco

**Affiliations:** 1 Istituto Nazionale di Geofisica e Vulcanologia, sezione di Bologna, Bologna, Italy; 2 INAF/IAPS, Rome, Italy; 3 Istituto Nazionale di Geofisica e Vulcanologia, sezione di Pisa, Pisa, Italy; 4 Dipartimento di Fisica, Università di Torino, Torino, Italy; 5 INAF – Osservatorio Astrofisico di Torino, Pino Torinese (TO), Italy; 6 Istituto Nazionale di Geofisica e Vulcanologia, sede di Napoli, Napoli, Italy; 7 Department of Earth Sciences, University of Florence, Florence, Italy; 8 CNR-IBE – Institute of Bioeconomy – National Research Council, Sesto Fiorentino, Italy; 9 LaMMA Consortium, Sesto Fiorentino, Italy

**Keywords:** Seismology, Meteoritics

## Abstract

On March 5, 2022, a 12 kg meteoroid crossed the sky above Central Italy and was observed by three different observational systems: the PRISMA all-sky camera network (10 stations), the Italian national seismic network (61 stations), and a 4-element infrasound array. The corresponding datasets, each with its own resolution, provided three independent assessments of the trajectory, size and speed of the meteoroid. The bolide traveled across central Italy with an azimuth of 102 degrees, becoming visible at about 91 km above sea level with a velocity of about 15.4 km/s. Its visible trajectory lasted about 15 s. Reasonably, the residual portion of the ablated bolide terminated its path in the Adriatic Sea and could not be recovered. Seismic and infrasound data well match optical observations detecting the bolide Mach cone at 68 km above sea level with a back azimuth of 25 degrees with respect to the array. By comparing results from the three different systems, discrepancies are within the estimated uncertainties, thus confirming the mutual consistency of the adopted methodologies. Therefore, this study shows that different approaches can be integrated to improve the detection capability for bolide crossing the sky in monitored regions.

## Introduction

On March 5, 2022, 18:55 UTC, Central Italy sky was crossed by a very bright fireball. Several media reports from different cities captured the attention of different research groups whose sensors have recorded the signature of this bolide passage.

Fireball observation is not a rare occurrence and scientific literature includes studies that discuss and model various data observations^1–4^.

Different monitoring systems exist, based on geostationary satellites as for the case of GLM (Geostationary Lightning Mapper) operated by NASA^5^ or by grounded networks of camera systems whose activity was initiated after the first successful meteorite fall observation and recovery in Czechoslovakia^6^ and the establishment of the European Fireball Network^7^. This first success gave rise to the birth of many other networks worldwide, such as the Prairie Meteorite network^8^, the Meteorite Observation and Recovery Project^9^, the Southern Ontario All-Sky Meteor^10^, the NASA All Sky Fireball Network^11^ and the Desert Fireball Network^12^ among others.

Since 2016, the Italian territory benefits of the all-sky camera network PRISMA (*Prima Rete Italiana per la Sorveglianza sistematica di Meteore e Atmosfera*) operated by Istituto Nazionale di Astrofisica (INAF). This is the first Italian network for meteor and atmosphere systematic surveillance. This network continuously monitors the sky, and it can record bolides down to a visual magnitude of -2 (-1 in very dark sites).

This can be done because, while entering the denser parts of the atmosphere, the meteoroid is heated up very quickly and when the surface temperature reaches about 2200 K the meteoroid material starts to sublimate from the surface and fills the surroundings of the body with its hot vapors. Meteor light consists mostly of radiation of discrete emission spectral lines belonging for the most part to metals and mainly to iron^13^.

As discussed in detail in the following sections, meteor light can provide an accurate determination of the speed and trajectory of such bright meteors^14,15^. PRISMA is a partner of the international collaboration FRIPON (Fireball Recovery and InterPlanetary Observation Network)^16–18^. Currently PRISMA operates more than 60 all-sky cameras over the Italian territory with the aim of recovering fragments fallen on ground (meteorites) and to create a database of bright meteors events. Since 2017, two meteorites were recovered on the Italian soil thanks to the observations and data analysis of PRISMA, i.e., Cavezzo on January 1st, 2020^12^ and Matera (provisional name) on February 14, 2023. For these two cases, the scientific team of PRISMA computed a detail picture of the two events among which: precise trajectory, estimated of initial and final mass of the meteoroid/meteorite, orbit, and area of probable fall (strewn field). To maximize the recovery efficiency of meteorites, the PRISMA team adopted an innovative approach by informing and involving the local population in the on-field search, providing them with the computed strewn field area. These are two out of less than 50 meteorites recovered since 1959 so far at global scale, thanks to existing fireball networks^19,20^.

Other types of monitoring networks exist, and they can capture different signals generated by massive objects entering the atmosphere at speed. This is the case of infrasound arrays or seismic networks that provide a different observation point for such an event (e.g.,^21^). Seismometers record the ground shaking associated with earthquake sources or other phenomena either of natural or anthropogenic origin. In Italy, the Italian Seismic Network^22^ densely covers the entire country with about 500 stations that are used to monitor the seismicity occurring within the national territory and abroad. Data is acquired, archived, and shared in real-time through the INGV (Istituto Nazionale di Geofisica e Vulcanologia) node for EIDA (European Integrated Data Archives)^23^. Given the high sensitivity and the quiet location of most of the seismic stations, tiny signals as very small earthquakes (down to magnitude 0 and below in some regions) can be detected, but the network also catches the seismic signature of other events both natural and anthropic as quarry blasts^24^ and storms^25^ but also human activities and their quietening of human activities^26^.

For the case of fireballs crossing the sky, a seismic network can record two types of signals. The first is the shock wave generated by a body that enters and crosses the atmosphere at a velocity faster than the speed of sound (e.g.,^4,19^). The second, concerns the case when such a body explodes and generates a point source pressure wave (e.g.,^27,28^). In both cases, seismometers register the coupling of the atmospheric pressure wave with the ground. For large bodies that fall on the ground with sufficient momentum, seismometers would also record the impact itself (e.g.,^29^).

**Figure 1:**
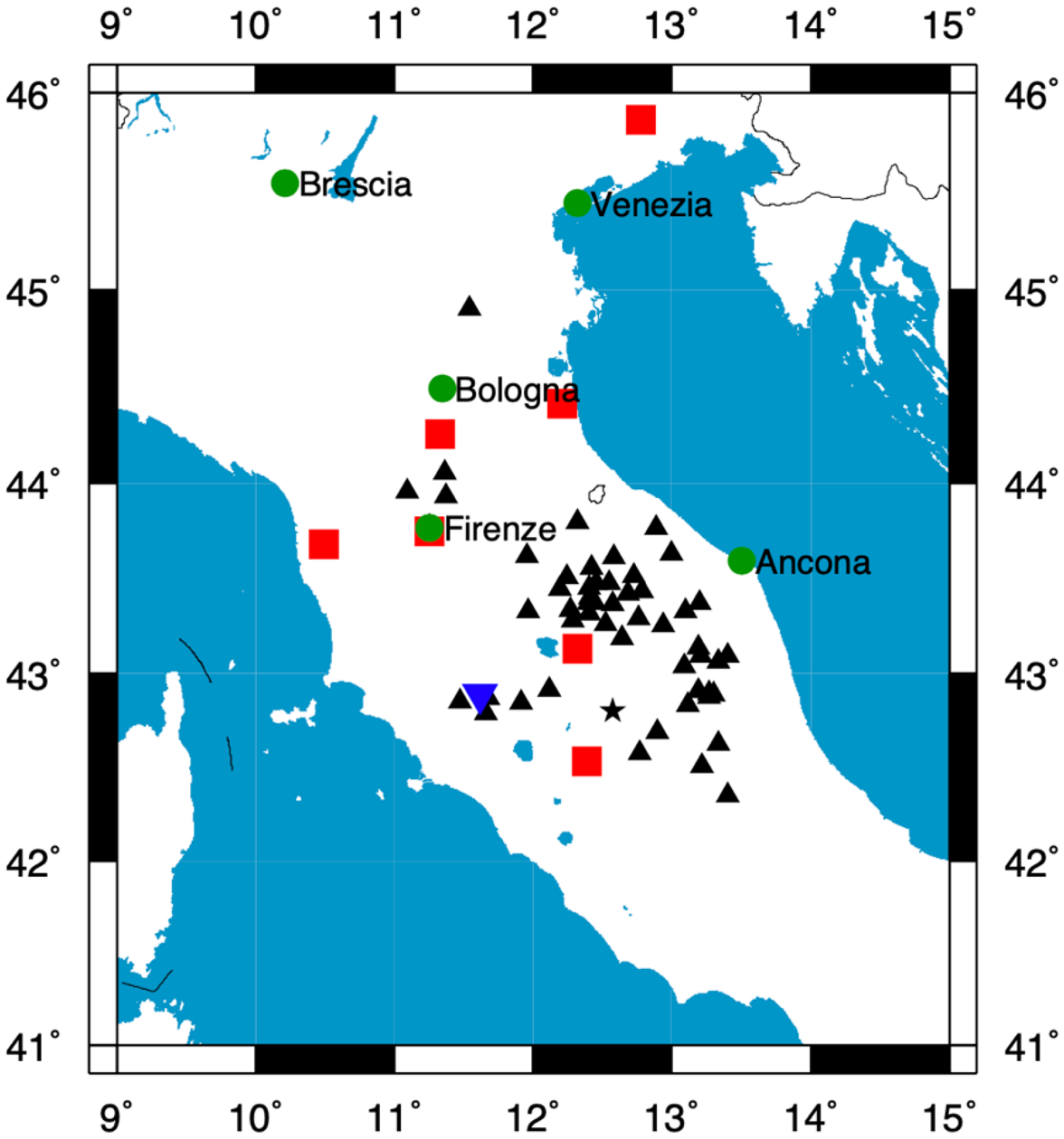
Map of the region where the passage of the bolide was observed. Green circles identify the major town from where witnesses were reported by the media. Black triangles are seismic stations apart from station MOMA (black star). Finally, red squares are a portion of the cameras belonging to PRISMA networks that detected it, and reverse blue triangle is the location of the infrasound array. Blue reverse triangle is the location of the infrasound array on Mount Amiata. Map was generated using Generic Mapping Tools software, version 4.5.18 https://www.generic-mapping-tools.org/).

The pressure wave generated by bolides traveling faster than the speed of sound or bolide explosions can also be captured by infrasonic microphone arrays. This type of arrays is commonly used to detect explosions as it is the case of the International Monitoring System operated in the framework of the Comprehensive Ban Treaty (CTBT) for nuclear tests at global scale^30^. In addition, small-aperture infrasonic arrays are widely used for volcano monitoring activities^31^. In March 2022, an infrasound array, belonging to the University of Firenze, was operational on Mount Amiata (Southern Tuscany, Fig. 1). This array was used to monitor regional or global scale large volcanic eruptions^32^.

Aim of this work is a multidisciplinary analysis of the different data that recorded this bolide passage with the objective of probing the different detection and location capabilities and to test theoretical wave propagation models as well as velocity profiles in the stratosphere and below. To our knowledge, this is the first time camera image recordings of the bolide passage are integrated with seismic and infrasound observations. Among the scientific interest in the nature, size and composition of specific objects, this type of event also provides unconventional seismic data, and this gives the occasion to test different methods and to define the seismic signature of such events. One of the outcomes could also be the automatic search of previous records by means of machine learning or template matching techniques and the implementation of tools that recognize forthcoming events as soon as they occur.

The speed of an extraterrestrial body entering the atmosphere can range between 11 and 72 km/s, well above the speed of sound that ranges between 280 and 330 m/s depending on the height above sea level. The fireball motion in the atmosphere produces a Mach cone^33^ which is characterized by an abrupt, nearly discontinuous change in pressure, temperature, and density of the medium. Mach cone is a near cylindrical cavity which propagates outward at supersonic speeds (initially) from the meteoroid path. For fireballs moving in the atmosphere, the Mach cone will have a small angle and can be effectively approximated as a line source^13^.

In addition to the cylindrical (or ballistic) shock produced by all meteoroids traversing the atmosphere, a fireball may also fragment during its flight^34^. This fragmentation produces a sudden increase in the rate of energy deposition and results in light flares and the associated quasi-spherical shock, independent of the Mach cone. All components of the low-frequency sound produced by the fireball representing the shock wave decaying at long ranges from the trajectory can be measured as infrasound on seismometers (when the sound couples to the solid earth) or directly by infrasonic arrays^29,35^.

Sound waves propagate through compressible media as the atmosphere. The propagation also includes refraction, attenuation and reflection that strongly depend on the properties of the air, mainly density, wind, and temperature gradients. Moreover, the speed of the sound waves is strictly related to density and pressure relationship, the direction of the waves is affected both by the density and the air motion (wind), that can cause the sound signal to bend. The Stratosphere and Troposphere are media characterized by strong thermal gradients and significant wind speed variations; therefore, they can substantially modify the propagation of sound waves.

This introductory section is followed by section Result in which we present observational data, and we show the results of our analysis and by section Discussion in which the forementioned results are compared and discussed.

## Results

The meteoroid entry into the atmosphere the evening of 5th March 2022 became visible at 18:55:45 UTC when it has been recorded by 10 cameras of the PRISMA network (see Fig. 1). Data from these stations are collected and combined to triangulate the meteoroid visible trajectory, to derive the main bolide parameters by application of suitable physical models, and to recover the meteoroid orbit prior to the atmospheric entry^19,36,37^. Table 1 shows the parameters computed: time and height at different steps.

**Table 1:** List of the most prominent flares recognizable in Fig. 3 and marked in Fig. 2.

Label	Time (UTC)	Height (km)
A	18:55:53.9	56.0
B	18:55:55.0	50.5
C	18:55:57.1	42.5
D	18:55:57.3	41.5
E	18:55:57.8	40.0
F	18:55:58.5	37.5
G	18:55:59.3	35.0

**Figure 2:**
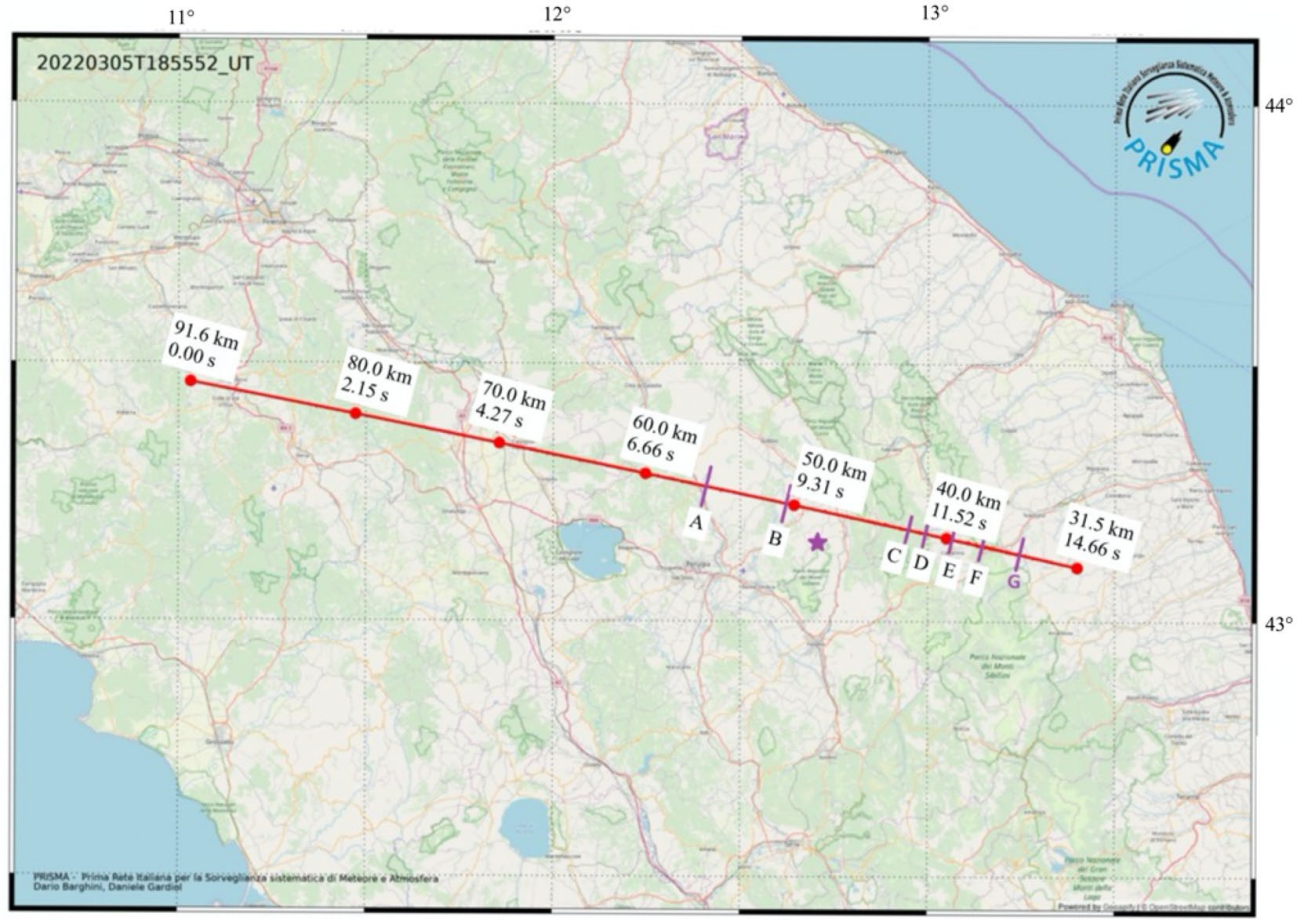
Ground projection of luminous part of the atmospheric trajectory of the meteoroid. Red markers reference different steps for which the relative time since its first detection, and the corresponding height is computed. Purple ticks with labels mark the position of the different flares listed in Table 1. Map was generated using IDL (Interactive Data Language), version 8, by NV5 Geospatial Solutions, Inc.; 2023, https://www.nv5geospatialsoftware.com/Products/IDL. Background map data copyrighted OpenStreetMap contributors and available from https://www.openstreetmap.org.

**Figure 3:**
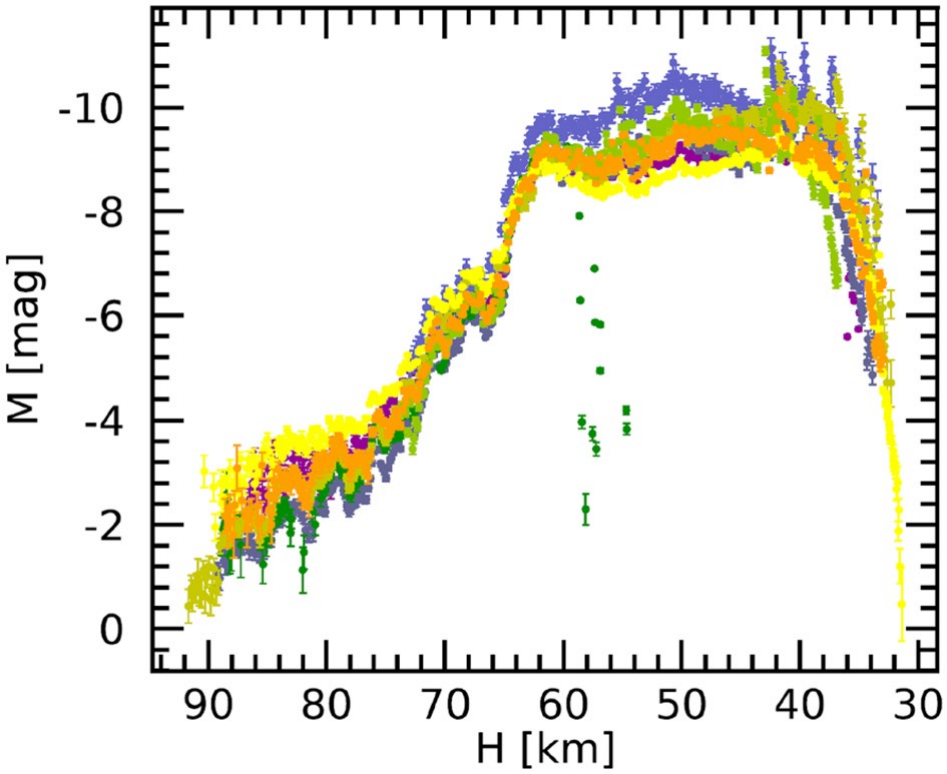
Absolute magnitude versus height as measured by the 10 PRISMA cameras. Different colors indicate observations by the ten PRISMA cameras. The dark green dots between 60 and 55 km are outliers of the brightness measure due to terminal part of the bolide’s trajectory going through a cloudy portion of the field of view of that particular camera.

The luminous phenomenon started at 91.22 ± 0.01 km above sea level with coordinates (43.462°, 11.022°), entering the atmosphere at a velocity of 15.5 ± 0.1 km/s, and its visibility terminated after 14.8 s at approximately 31.5 ± 0.1 km of height, coordinates (43.107°, 13.3904°) when the velocity was reduced to 6.4 ± 0.2 km/s (Fig. 2). The low inclination with respect to the horizon, around 16.5 degrees, made possible the quite remarkable visible atmospheric trajectory whose length is (210.0 ± 0.1) km and azimuth of about 101 degrees. This is the longest path ever observed by PRISMA network since its deployment. The absolute visual brightness of the bolide reached a plateau at approximately magnitude –10 that lasted for 6 s (Fig. 3). Assuming a typical chondritic bulk density of the meteoroid of 3.3 ± 0.2 g/cm^3^^38^, the estimated initial mass of the meteoroid was 12 ± 4 kg, corresponding to an equivalent diameter of 19 ± 2 cm. The final mass and diameter have been estimated respectively as 1.2 ± 0.4 kg and 9 ± 1 cm. Several flares (at least eight) can be recognized in the camera's recordings. These are also confirmed by the magnitude brightness versus height plot in Fig. 3 that sets a height of the first one at about 56 km. Flares become more intense and frequent from 43 km (position marked in Fig. 2). Figure 3 also highlights a sudden increase in absolute brightness between 70 and 65 km of height that will be later discussed.

**Figure 4:**
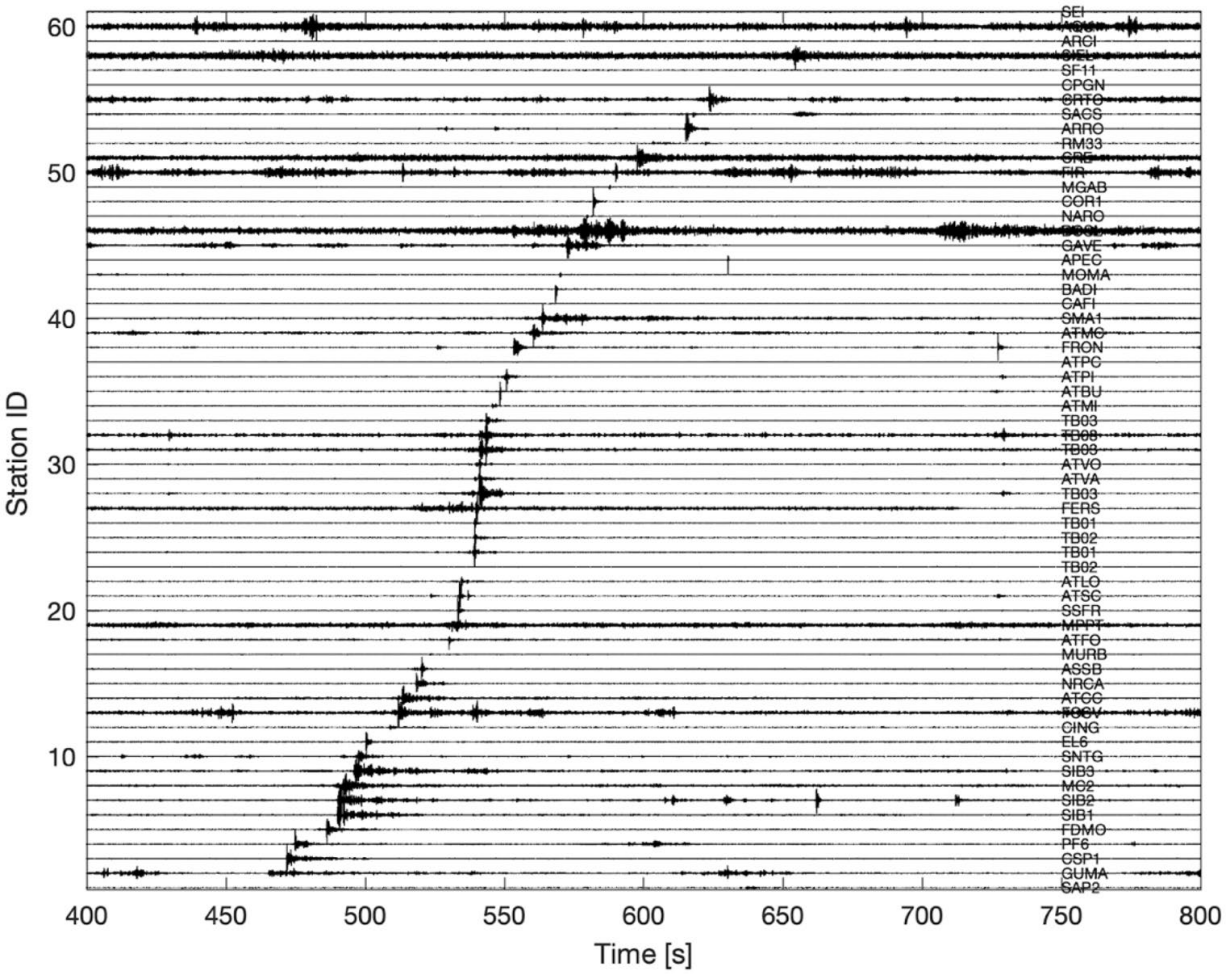
Record section showing the progressive arrival of the transient across the Italian permanent seismic network. Time is expressed in seconds since March 5, 2022, 18:50 (UTC). Station names are reported on the right side of each seismic record.

We then inspected the INGV seismic stations operating in the area for the day of the event. A clear and coherent transient pulse consistent with the time of the fireball passage emerges above the background noise at 61 stations (Fig. 4). Time–frequency domain analysis reveals that a large part of the energy recorded by seismometers concentrates between 1 and 20 Hz. A band pass filter in this frequency band is then applied to raw data to enhance the signal-to-noise ratio. This helped to observe: (i) the lack of clear direct body wave phase as. the P-wave, a pressure wave that vibrate in the direction of propagation in solids, fluids and gases, (ii) a very short duration of the wave train (< 5 s), (iii) waveform similarity in terms of duration and amplitude among the selected stations, (iv) an apparent propagation velocity significantly smaller than that expected for seismic body waves. The combination of these four pieces of evidence (no clear body wave onset, short duration, the lack of spatial attenuation, low propagation velocity) suggests the incompatibility with earthquake-like source.

As recognizable in Fig. S1, a low frequency ringing (1–3 Hz) lasting for about 5 s that precedes the high frequency arrival by a few seconds is clearly visible at some seismic stations. These are Rayleigh waves originated by the acoustic-to-seismic coupling as observed by other authors^21,39^.

Following the observations from the PRISMA network, we tested two hypotheses as sources of the high frequency part of the seismic signal: the Mach cone generated by the entrance and passage into the atmosphere with the explosion of the meteoroid itself.

The latter was discarded since the observed arrival times at the selected 61 stations did not fit with the theoretical travel times of an explosive point source propagating at the speed of sound (~ 300 m/s). Conversely, we could estimate an average apparent speed of propagation ${v}_{a}$ of about 1100 m/s (Mach 3.5).

The distribution of the arrival times of the transients observed at the seismic stations is compatible with a source moving along the path of the fireball suggesting that this transient could originate from the coupling between the shockwave generated by the fireball (Mach cone) and the Earth surface in proximity of each seismic station (Fig. S2 in the Supplementary material). To support this hypothesis, we model the Mach cone propagation using the trajectory described in Fig. 2. This was discretized in terms of altitude, position, and speed and at each time step of the trajectory, we generated a spherical wave front which propagates in a homogeneous medium with an average speed of 295 m/s.

**Figure 5:**
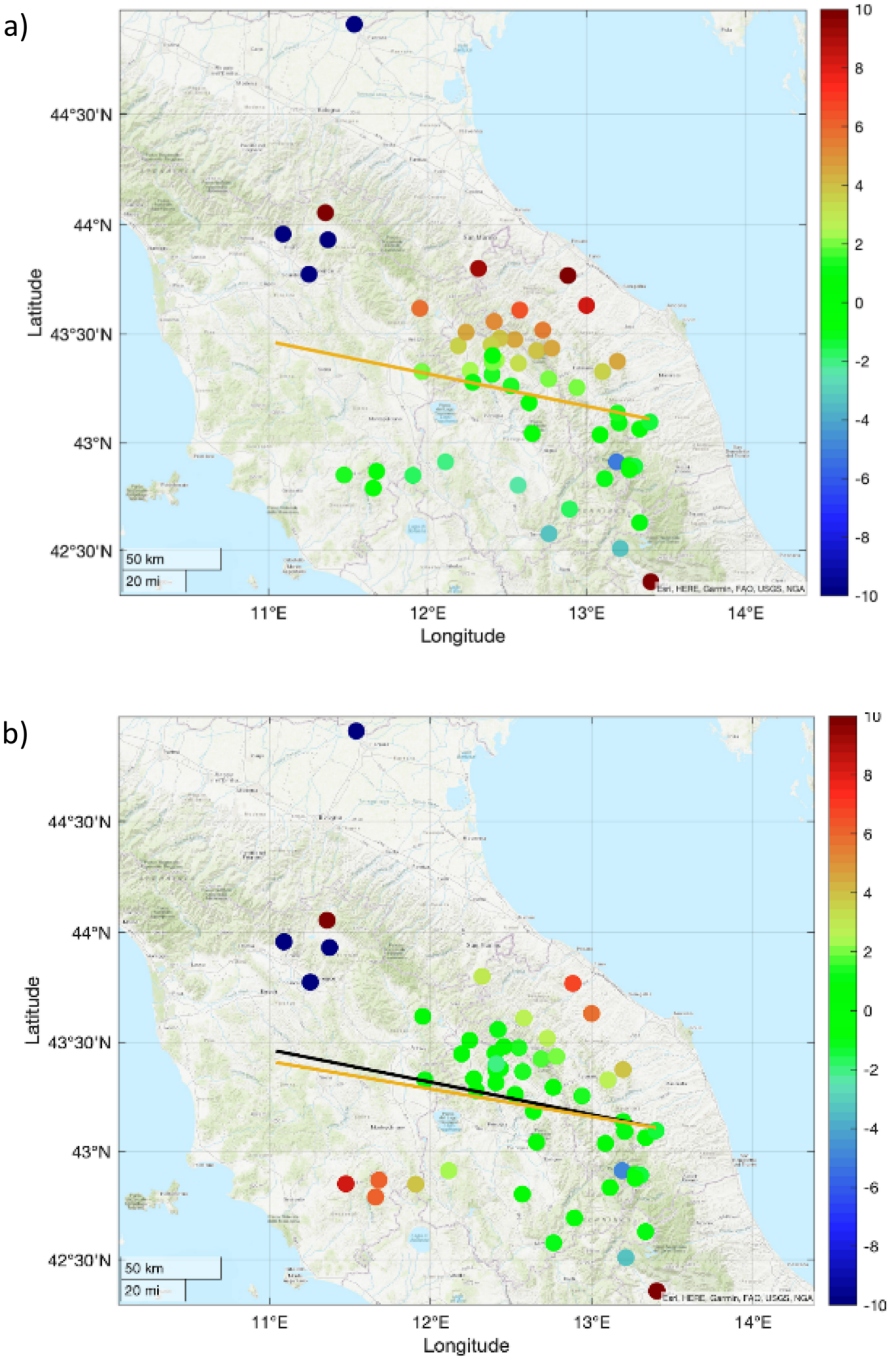
Map of the difference between theoretical and observed travel time for the case of Mach cone propagating along the meteoroid path and then coupling with ground. Frame (**a**) shows the residuals calculated according to the trajectory observed by Prisma network (orange solid line). In frame (**b**) the orange solid line represents the trajectory which minimizes the residuals. The angle between the black (observed) and orange lines (rotated trajectories) is 12 degrees toward the south. Circles are color coded according to the residuals (expressed in seconds). Maps were produced using MATLAB version: 9.13.0 (R2022b), Natick, Massachusetts: The MathWorks Inc.; 2022 https://it.mathworks.com/products/matlab.html.

The theoretical times of the wave front arrival calculated for each station are used to compute the residual shown in Fig. 5.

Residuals are not randomly distributed around zero, but two clusters can be recognized. The first, is associated with stations located south of the bolide’s trajectory, and it is mostly characterized by positive residuals. Conversely, the region north or the trajectory mostly exhibits negative residuals (Fig. 5a). This indicates systematic prediction error that we attribute to the action of wind at high altitudes, that would move the air mass in turn causing a rotation of the Mach cone. The preferred angle with respect to the PRISMA solution that minimizes the residuals results in a 6 degrees rotation toward south of the meteoroid path (Fig. 5b). During the passage, the meteoroid crossed a portion of the atmosphere characterized by strong northerly winds. The available reanalysis data used in this study indicate, between 47 and 20 km in height, winds with a prevailing northern component with strong directional (anticyclonic) and intensity shear. At the isobaric height of 1 hPa (about 47 km) the Mach cone intersected north-westerly winds blowing between 56 and 58 m/s, thus undergoing a first substantial deviation from the theoretical path. At the isobaric height of 10 hPa (about 30 km) the Mach cone encountered winds between 19 and 25 m/s coming from N-NW, finally at 20–30 hPa (around 24–26 km) north-easterly winds at 6–12 m/s. The wind pattern in the upper and middle stratosphere is compatible with a southward shift of the Mach cone trajectory, especially in the initial and middle part of the bolide passage.

**Figure 6:**
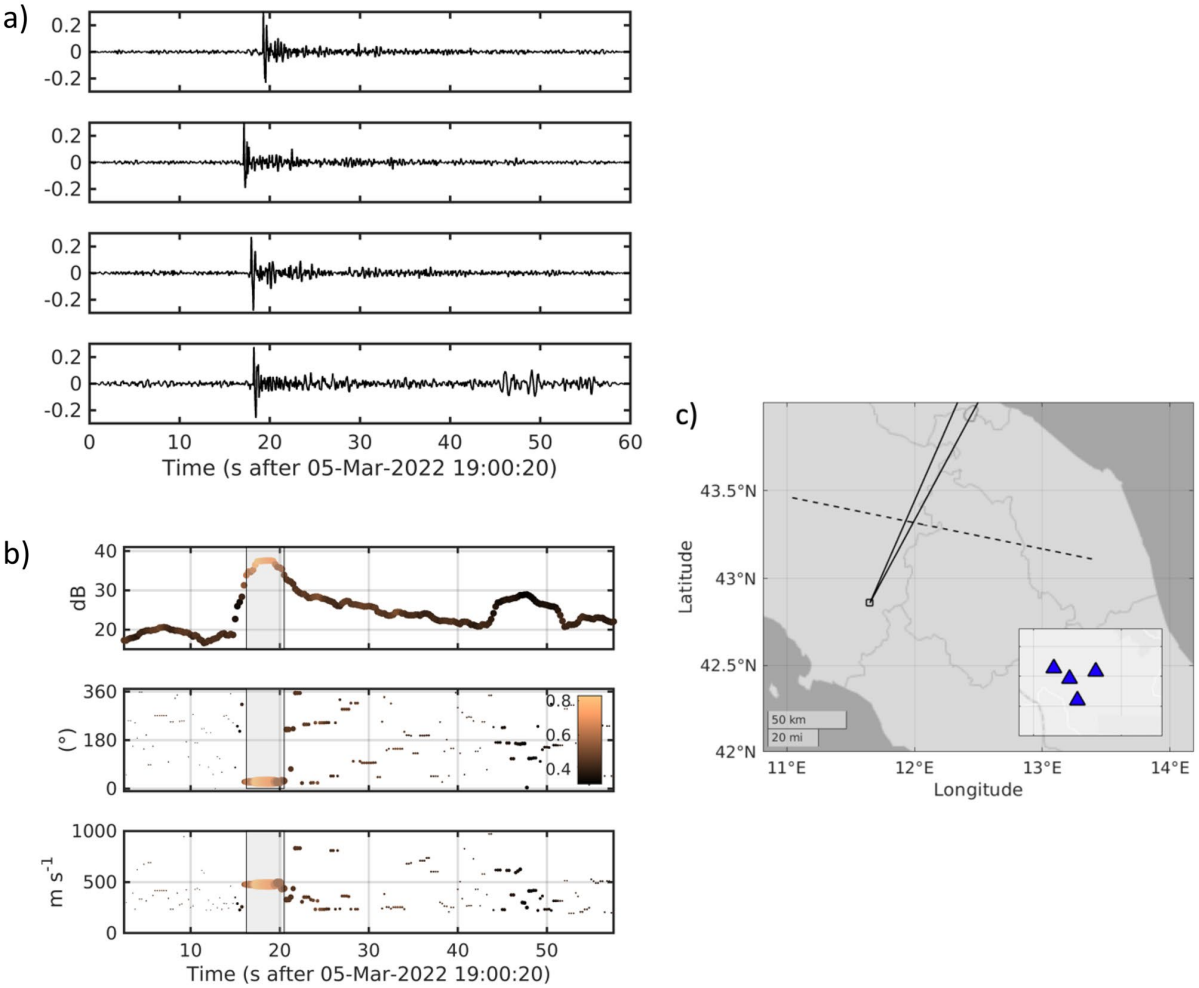
(**a**) recordings of acoustic pressure at the infrasonic array displayed in panel (**c**). The y-axis unit is in Pa. (**b**) Results from beam-forming analysis. From top to bottom, the different panels illustrate the temporal evolution of the beam-power, back azimuth to the source, apparent velocity. Symbols are sized according to the beam power, and colored as a function of the multichannel coherence, according to the color scale reported in the middle panel. The shaded patches mark the region for which the beam power is larger than 50% of the largest power. (**c**) Location of the infrasonic array in central Italy; the azimuthal wedge corresponds to the range of back azimuth selected from the middle plot in panel (**b**). The inset shows the geometry of the array.

This also produced a clear pulse-like signal in the recordings of the infrasonic array located in Central Italy (Fig. 6). The pulse unveils a peak pressure on the order of 0.33 Pa (Fig. 6a). Using a conventional multichannel processing technique (see Methods Section), we estimated a back azimuth to the source on the order of 25° (+ /−1°) N, and an apparent velocity of about 480 m/s. Assuming a local speed of sound at the array of 336 m/s, this indicates that the acoustic wavefront impinges at the array with an angle of about 46° with respect to the horizontal. For these back azimuth and incidence angles, a straight ray would intersect the horizontal trace of the bolide trajectory at the coordinates (43.3244°, 11.9574°), corresponding to an elevation of about 68 km above sea level. For that source location, we derive the trajectory of an acoustic wave traveling in a standard stratified atmosphere (http://www.pdas.com). From this forward calculation, we find an expected incidence angle of 41°, which compares well with the observed one. The deviation of the observed acoustic beam from the shortest path (i.e., the line perpendicular to the Mach cone) provides an indication of the ray path distortion due to lateral variation in the acoustic velocity and the wind action.

## Discussion

We used three different datasets that recorded the bolide passage on March 5 2022, to carry out a multi-messenger analysis of the track, to better understand the different aspects of the passage itself, of the different source of signals and on how these can support each other to provide the best model and to enhance the detection capabilities. The most relevant result is that each one with the specific resolution capacity, the three datasets provide consistent results intercepting the trajectory.

The infrasonic array could determine, for the acoustic signal, back azimuth, and incidence angles of 25 and 46 degrees, respectively. The non-orthogonality of the observed back azimuth with the optically reconstructed trajectory shown in Fig. 6 is motivated by the fact that the recorded signal propagates from the bolide following the Mach cone shape and not as a simple spherical wavefront. To check the consistency of this observation with the optical one, we note that the back azimuth intercepts the optical trajectory at a height of about 68.7 km. The discrepancy between the computed and true incident angle results to be 5 degrees smaller, proving that the two tracking approaches are mutually consistent.

The seismic analysis appears more complex, and results are less constrained since recorded signals come from the conversion of the sound wave hitting the ground and this limits the back projection of the traveled path. This multi-disciplinary stage was also the occasion to set up a methodology to model seismic signature of bolide passage. Results are encouraging and the resulting direction of propagation appears consistent with the optical one although wind speed uncertainty at high altitude provides large indetermination in the input velocity model.

Ideally, when the sound wave generated by the Mach cone is recorded by a significant number of seismic stations, the detected travel times could be used to back trace the position and motion of the fireball along its track. To this aim, the back azimuth of the incoming sound wave at each station should be known and this must rely on a reliable atmospheric model, including pressure and density of the air layer and wind speed. For this specific case, we did make tests, by computing Finite Differences sound wavefield in a realistic atmospheric model, assuming the pressure, temperature, and wind model of the 50–70 km above sea level, as provided by ERA5 reanalysis. However, with respect to the travel times computed for a homogeneous model, the resulting travel times give much worse fit to the data. This indicates that the available atmospheric model is not precise enough for computing sound wave travel times, at least for this specific case. Considering the above, we adopted a homogeneous atmosphere, providing acceptable time residuals.

In this framework, we also attempted the analysis of the flares spotted by optical cameras, but no clear signal could be detected on the seismic waveforms at the relevant theoretical travel times. This can motivate from the fact that observed flares were not associated to a significant pressure wave.

We note that PRISMA network is composed of optical cameras, and it could be difficult to capture bolide passages in daytime or when the sky is covered by clouds. Conversely, seismic stations and infrasonic sensors can record such signals over 24 h although, given the small amplitude of the expected signals, their detection capability can be limited by poor signal-to-noise conditions. Moreover, anthropogenic, or natural transient with similar duration and frequency content could generate false detection. In conclusion, the results of this analysis evidence the potential for a multi-messenger approach to bolide observation that could help to improve the detection capability, densify the event catalogue and help to discriminate coherent but anomalous signals on seismic stations. It remains open the possibility of locating the trajectory of the bolide by only using seismic network observation when temperature, pressure and wind profile are weakly constrained.

## Methods

### Optical data

Each PRISMA station autonomously acquires images at a rate of 30 frames per second (fps) and triggers for the passage of bright meteors in its field of view (FoV), looking for a moving source by a simple frame difference method^40^. At the same time, each camera records longer exposure images every ten minutes during night-time to capture reference stars (up to magnitude + 4) for calibration purposes. Data collection from each camera is managed by the FRIPON central server, located at the LAM/OSU ((Laboratoire d’Astrophysique de Marseille/Observatoires des Sciences de l’Univers) Pythéas facilities in Marseille^41^ and a copy of the PRISMA Italian data is synced to the servers of the INAF-IA2, in Trieste.

The first task in the analysis of such optical data is the definition of the astrometric (positional) and photometric (intensity) calibration of each camera. This is done by comparing the apparent position and intensity of stars detected in the FoV with their catalogue coordinates and apparent magnitude^36^. Calibration results over monthly statistics of stars for each camera are therefore used to reduce the detection videos of the same event, to determine the apparent path of the meteor in equatorial coordinates as seen by each PRISMA cameras that registered the meteor. Data are combined to determine the three-dimensional atmospheric trajectory of the event with the triangulation method^33,34^ To do so, we use the lines-of-sight method by^40^ consisting in minimizing distance residuals between each observation from each camera and the straight-line trajectory to be determined.

The triangulation processing allows to compute the speed and absolute magnitude profile of the meteor as a function of time. These data are finally used to evaluate a physical model which allows interpreting the dynamic evolution of the event and deducing the main physical parameters of the meteoroid^18,42,43^. Such physical model encompasses the description of the phenomena of deceleration due to atmospheric drag, ablation (mass loss) and light emission, enclosed in the following system of differential equations:

$$\left\{\begin{array}{c}\frac{dH}{dt}= -V\mathrm{sin}\gamma \\ M\frac{dV}{dt}= -\Gamma S{\rho }_{a}{V}^{2}\\ \frac{dM}{dt}=\sigma MV\frac{dV}{dt}\\ I= -\tau \frac{d}{dt}\left(\frac{1}{2}M{V}^{2}\right)\end{array}\right.$$

where $H$ is the altitude of the meteor above the ground, $V$ is the apparent speed module of the meteoroid, $M$ is the mass of the meteoroid, $S$ is the area of its cross-section and $I$ is the light intensity emitted by the meteor (as a function of the time $t$ since the beginning of the event). The other quantities included in the equations’ system are the physical parameters of the meteoroid and the meteor, and they are the trajectory inclination with respect to the ground ($\gamma$), the atmospheric drag coefficient ($\Gamma$), the atmospheric density (${\rho }_{a}$), the ablation coefficient ($\sigma$) and the luminous efficiency ($\tau$). All these parameters are estimated by fitting this equations’ system to the observed altitude, speed, and magnitude data of the meteor event by a numerical integration approach, together with the starting values of the system which define the pre-atmospheric parameters, such as the pre-atmospheric mass (${M}_{\infty }$) and speed (${V}_{\infty }$).

Further details about the PRISMA data processing pipeline are given in^44^.

### Infrasonic data

Infrasonic data are from a 4-element array deployed on the southern flank of Mt. Amiata Volcano (Central Italy) and operated by the Geophysics Lab at the Earth Science Dept. of the University of Florence. The array has an aperture of about 1500 m, and its elements are located at an average elevation of about 1080 m. Each array element is equipped with a differential pressure transducer, with a sensitivity of 25 mV/Pa, that can detect atmospheric pressure fluctuations in the range of ± 100 Pa. The sensor has flat response between 0.01 and 20 Hz. Data are recorded continuously, at a rate of 50 samples per second. We analyzed the infrasonic signals using a conventional frequency-domain beamforming approach^38,39^ as implemented in the ObsPy libraries^45–47^. The analysis is conducted over the 1–10 Hz frequency interval, using 5-s-long windows sliding along the array recordings with 95% overlap. Under the plane-wave approximation, the method returns the two Cartesian components of horizontal slowness $[{s}_{x},{s}_{y}],$ from which one obtains the back azimuth to the source:

$${f}_{b}R = 180 + atan(\frac{{s}_{x}}{{s}_{y}})$$

and the apparent velocity

$${v}_{a} = \frac{1}{\surd ({{s}_{x}}^{2}+{{s}_{y}}^{2})}$$

The angle of incidence, i.e., the angle between the acoustic ray and the normal to the Earth’s surface, is finally obtained from the relationship:

$${f}_{i} = asin ( \frac{{v}_{s} }{{v}_{a}} )$$

where ${v}_{s}$ is the speed of sound at the array location.

### Seismic data

Seismic data used for the seismological analysis were recorded from stations belonging to the Italian National Seismic Network, the earthquake monitoring infrastructure managed by the Istituto Nazionale di Geofisica e Vulcanologia (INGV). We used data from a set of 61 stations surrounding the bolide’s trajectory (Fig. 1). Station metadata and continuous waveforms are distributed through the Orfeus European Integrated Data Archive (EIDA) federation and the International Federation of Digital Seismograph Networks (FDSN), using standard protocols under an open definition compliant license.

Since the operating seismic network is equipped mostly with broadband seismometers and 24-bit datalogger, we remove instrumental response from the seismic data recorded to obtain a homogeneous dataset. Data were analyzed in the time and frequency domain, then filtered in the 1–20 Hz band to remove the marine microseisms and enhance the observed transient traveling across the network.

A general characteristic of the observed transient is the presence of a negative energetic high frequency pulse. The high frequency pulse shape is known as the N-wave (with an inverted N letter) or W-wave when recorded in displacement or velocity units respectively (Fig. S3), with compressive first motion, and is observed for meteor events, sonic booms, atmospheric explosions, and thunder^48–50^. The abrupt shape of N-waves is inherited from the nearly instantaneous pressure change in the initial, nonlinear shock wave^29,51^.

We analyzed the velocity recordings and used the arrival time of the high frequency W-waves to the subsequent analyses, such as the estimate of the apparent velocity (i.e., the velocity with which a seismic-signal wavefront appears to travel along the surface of the Earth) propagation for the high frequency part of the transient which is of about 1200 m/s, considerably lower than the average velocity of body waves for the area.

A further characteristic, a weak, low frequency and nearly monochromatic signal is also clearly visible at some stations a few seconds before the most energetic high frequency arrival. Previous studies^20,37^ describe these low frequency phases preceding the main arrivals as Rayleigh waves originated by the acoustic-to-seismic coupling.

To test this hypothesis, we choose the waveform recorded at MOMA, the station that exhibits one of the largest low frequency arrivals. After a frequency analysis, we filtered the waveforms in the 0.5–4 Hz band to enhance the low frequency arrivals (Fig. S4) and then rotated the seismograms according to the expected back azimuth for the station MOMA to analyze the waveforms in the vertical-radial plane (Fig. S5).

From the obtained hodogram (bottom panel in Fig. S5) we can observe a retrograde sense of the ground particle motion as expected for a Rayleigh wave. Assuming a Rayleigh waves velocity of 2000 m/s we can estimate that the coupling between the pressure front and the ground occurs at an approximate distance of 10 km from the station.

### Supplementary Information

Supplementary Figures.

## Data Availability

PRISMA data can be accessed through the FRIPON portal https://fireball.fripon.org/. Seismic data are available at the Italian EIDA node http://eida.ingv.it. Seismo-acoustic data are available upon request to Maurizio Ripepe (Maurizio.ripepe@unifi.it).
