# Supplementary material for: The optical, seismic, and infrasound signature of the March 5 2022, bolide over Central Italy
Source: Sci Rep. 2023 Nov 30;13:21135. doi: 10.1038/s41598-023-48396-8 (PMC10689470; doi:10.1038/s41598-023-48396-8)
Supplement: Supplementary file 1 — Supplementary Figures. [file 41598_2023_48396_MOESM1_ESM.docx]

**The optical and seismic and infrasound signature of the March 5 2022, bolide over Central Italy**

Olivieri et al.

Supplementary Material


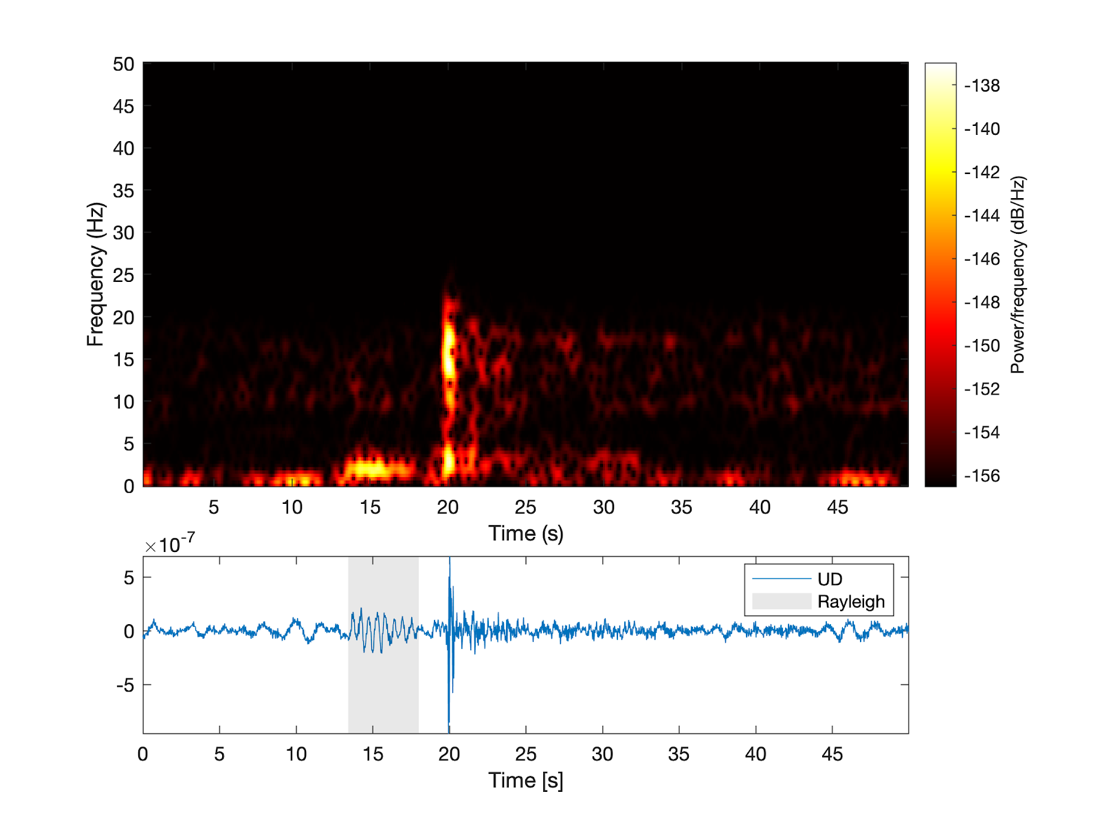


*Figure S1: Spectrogram (upper panel) and ground velocity time series (lower panel) for the transient recorded al MOMA station.*


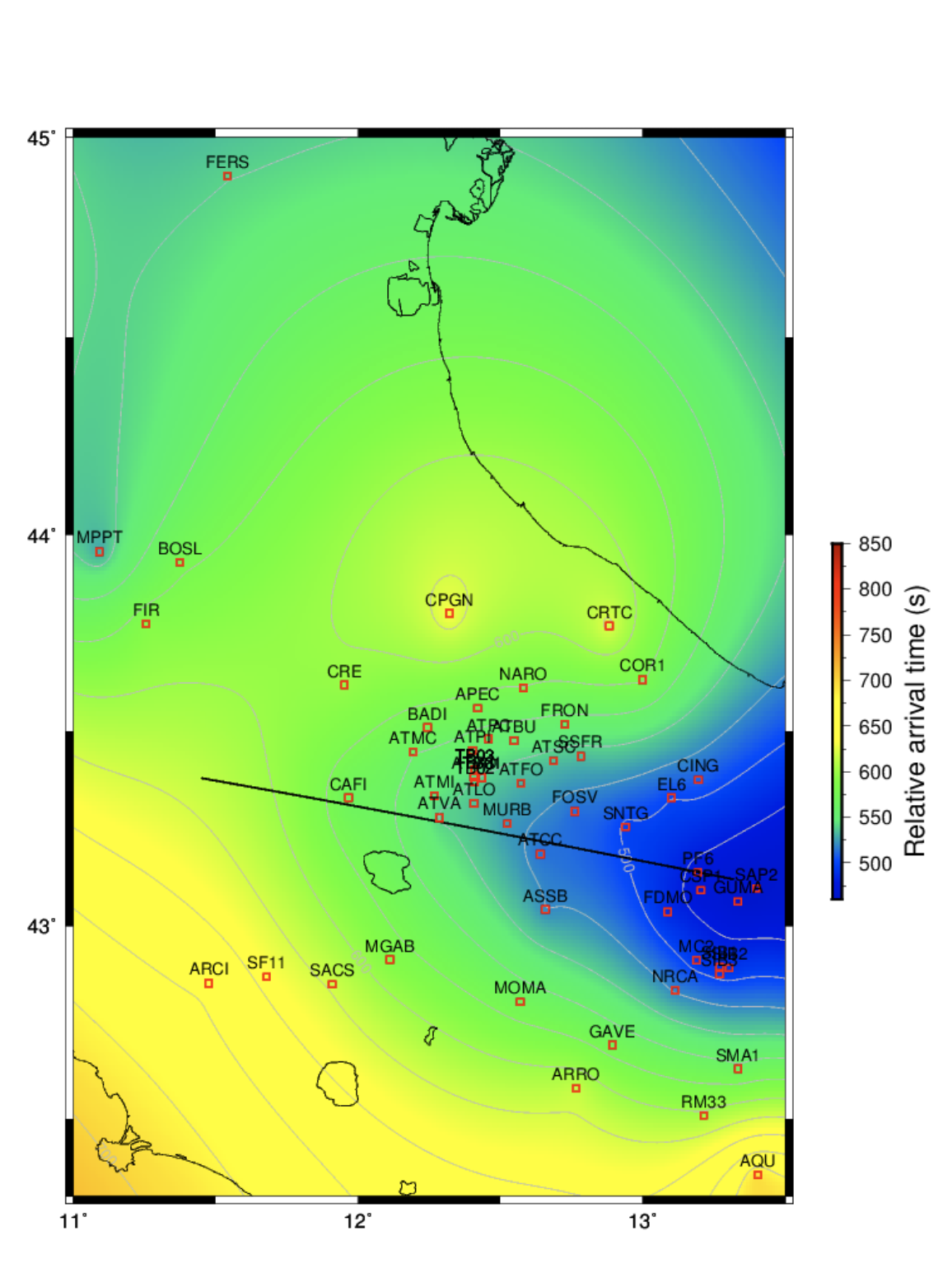


*Figure S2: Map of the high frequency transient arrivals. Black straight line indicates the bolide’s trajectory according to the PRISMA network. Time is expressed in seconds since March 5, 2022, 18:50 (UTC).*


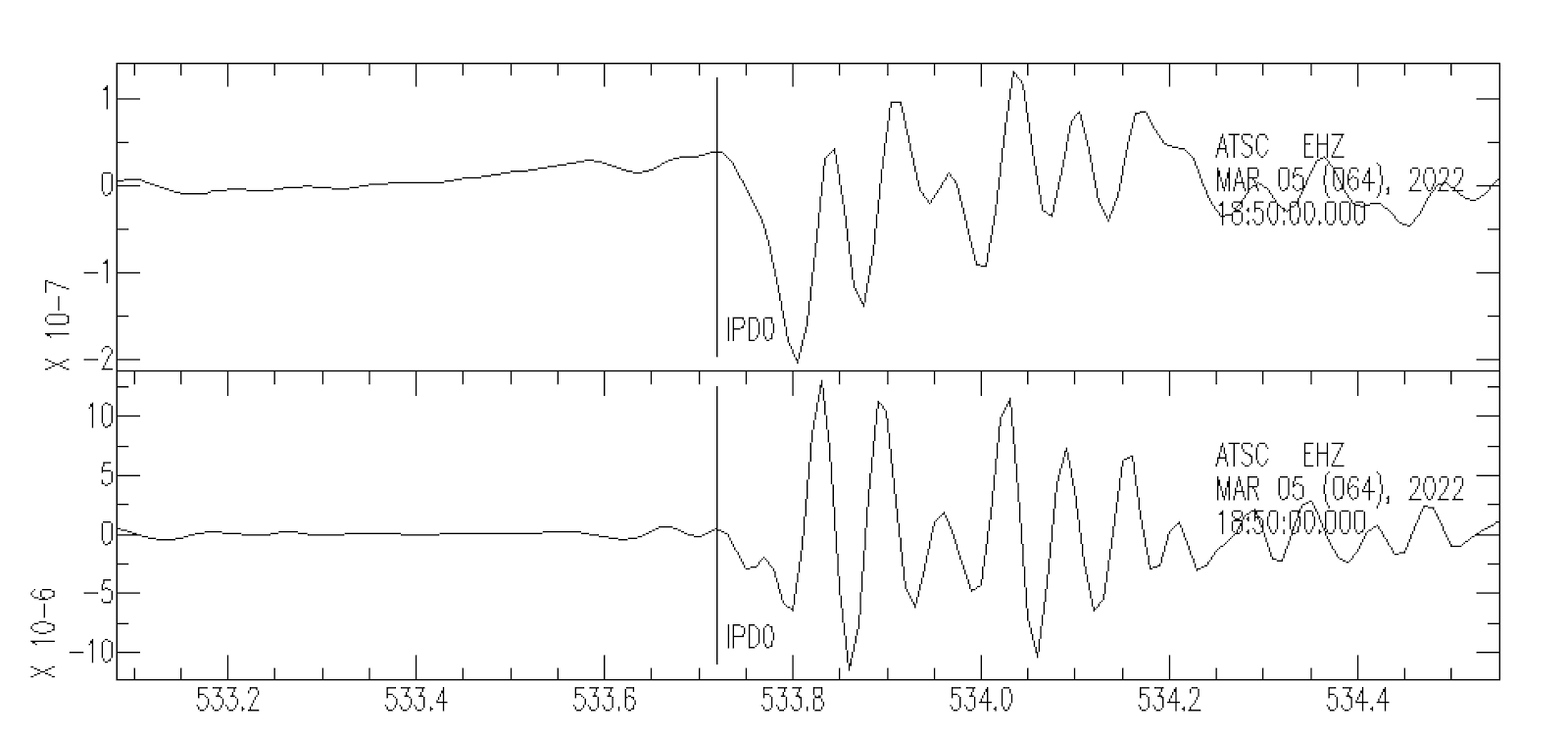


*Fig S3: Displacement (upper panel) and velocity (lower panel) time series for the station ATSC filtered between 1 and 20 Hz. The vertical bar indicates the arrival of N-wave and W-wave respectively.*


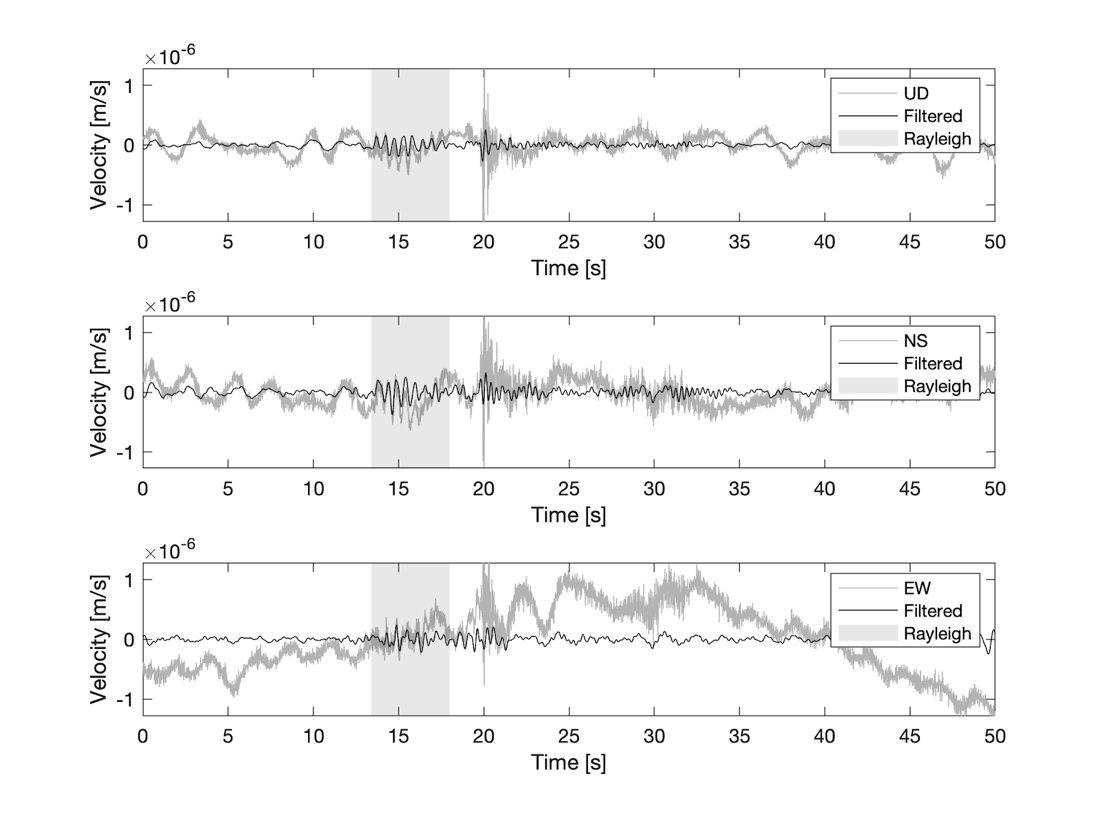


*Figure S4. Unfiltered (gray lines) and filtered (black lines) three component seismograms for the station MOMA. The gray band represents the seismogram portion reported in figure S5 which contains the observed Rayleigh waves.*


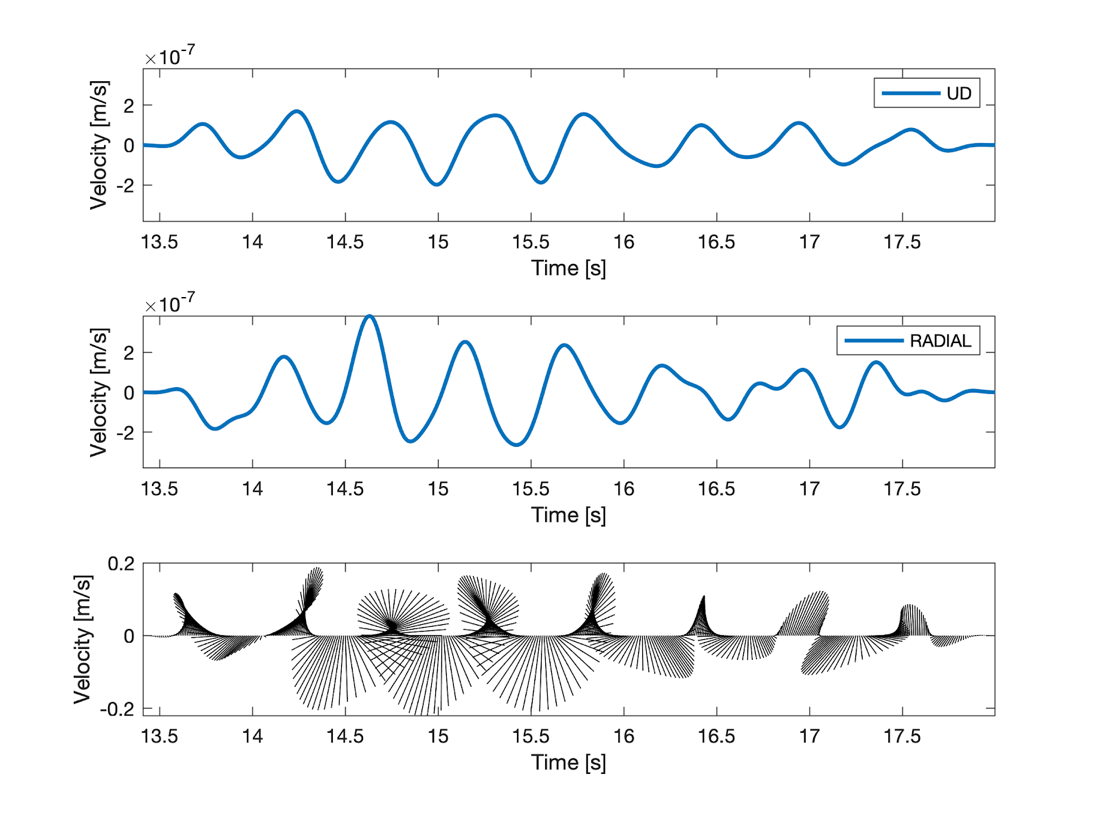


*Figure S5: in blue are reported the vertical and radial component of the ground velocity containing the observed Rayleigh waves. In the bottom panel hodogram, the vectors originating from each time step describe a retrograde sense of the ground particle motion.*
